# Transmission of *Grapevine leafroll-associated virus-1* (*Ampelovirus*) and *Grapevine virus A* (*Vitivirus*) by the Cottony Grape Scale, *Pulvinaria vitis* (Hemiptera: *Coccidae*)

**DOI:** 10.3390/v13102081

**Published:** 2021-10-15

**Authors:** Gérard Hommay, Antoine Alliaume, Catherine Reinbold, Etienne Herrbach

**Affiliations:** 1 UMR SVQV, Université de Strasbourg, INRAE, F-68000 Colmar, France; gerard.hommay@inrae.fr (G.H.); catherine.reinbold@inrae.fr (C.R.); 2 BioReval, F-80000 Amiens, France; contact.bioreval@gmail.com

**Keywords:** soft scale, *Coccidae*, *Closteroviridae*, *Betaflexiviridae*, *Vitis vinifera*

## Abstract

The cottony grape scale *Pulvinaria vitis* is a scale insect colonizing grapevine; however, its capacity as a vector of grapevine viruses is poorly known in comparison to other scale species that are vectors of viral species in the genera *Ampelovirus* and *Vitivirus*. The ability of *P. vitis* to transmit the ampeloviruses *Grapevine leafroll-associated viruses* [GLRaV]−1, −3, and −4, and the vitivirus *Grapevine virus A* (GVA), to healthy vine cuttings was assessed. The scale insects used originated from commercial vine plots located in Alsace, Eastern France. When nymphs sampled from leafroll-infected vineyard plants were transferred onto healthy cuttings, only one event of transmission was obtained. However, when laboratory-reared, non-viruliferous nymphs were allowed to acquire viruses under controlled conditions, both first and second instar nymphs derived from two vineyards were able to transmit GLRaV−1 and GVA. This is the first report of GLRaV−1 and GVA transmission from grapevine to grapevine by this species.

## 1. Introduction

**Figure 1:**
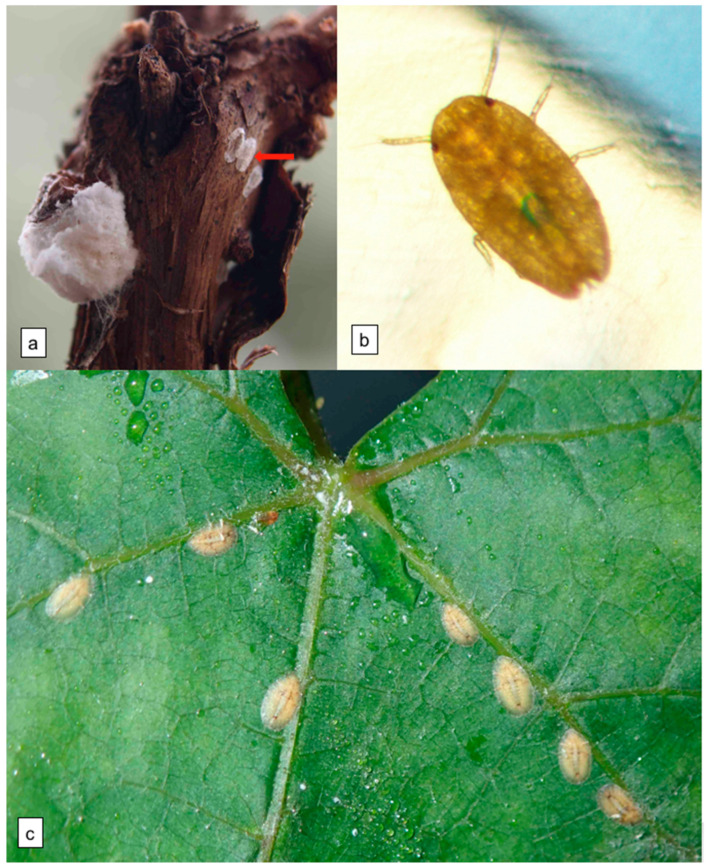
(**a**) *Pulvinaria vitis* specimens on a grapevine. Left: female with ovisac. Red arrow: three empty plate-like tests or “puparia” of males. (**b**) Second instar nymphs (L2) of *P. vitis* on a grapevine leaf, with honeydew drops. (**c**) Crawlers of *P. vitis* fed across a Parafilm^TM^ membrane stretched over a drop of purified virions added with methylene blue. Blue stain is visible in the gut of the nymph.

The cottony grape scale *Pulvinaria vitis* (L), family *Coccidae*, is a polyphagous species, which feeds on various woody plants (García Morales et al., 2016). It is usually scarce and dispersed in vineyards, but can sometimes proliferate and cause damage, weakening the vine by feeding on phloem sap and excreting honeydew on which sooty mould develops. Population outbreaks in vineyards have been reported in several countries [1,2,3,4]. *P. vitis* has also been reported as a pest on peach in Canada [5]. It is widely distributed in the Palearctic zone from western Europe to China, and southwards to the Mediterranean area as far as Iran [6]. It was introduced in North [1] and South America [7,8,9], and in New-Zealand [10,11]. *P. vitis* can reproduce sexually or parthenogenetically and develops one annual generation. Adult insects mate from September to October [3]. Adult females are 5–7 mm long, dark brown and convex, whereas adult males are winged and pinkish, only 1.5 mm long [6]. While males die after mating in the fall, females overwinter and then produce white waxy egg masses from the end of April to early June [3] (Figure 1a). As adults, females become sessile and each of them lays an average of 3500, though sometimes up to 5000, eggs [3], embedded within a cotton-like structure, called the ovisac, which grows beyond the shield and lifts it progressively. The eggs are orange to wine-red in colour and hatch from late May to June. The first instar nymphs (L1 or ‘crawlers’) are only 0.5 mm long, elongated oval in shape, with colour ranging from dark yellow to reddish-brown. The crawlers are very mobile prior to settling along leaf veins to pursue their development. *P. vitis* is dispersed by wind at any stage when it is active, but especially at the early crawler stage [5]. There are three nymphal stages, the first moult occurs in July and the second in August [3].

Grapevine leafroll disease (GLD) is one of the most important viral diseases of grapevines, caused by virus species in the family *Closteroviridae*. Among these, three species belonging to the genus *Ampelovirus* (*Grapevine leafroll-associated virus* [GLRaV]−1, −3, −4 strains 4, 5, 6 and 9) were shown to be transmitted by many species of mealybugs (*Pseudococcidae*) and soft scales (*Coccidae*) in different grapevine-growing regions worldwide [12,13], favouring GLD spread at a local scale. GLRaV−2, which belongs to the genus *Closterovirus*, has no known vector. Moreover, some of these scale insects are also vectors of three grapevine-infecting viruses associated with ‘rugose wood complex’ [13] assigned to the species *Grapevine virus A* (GVA), GVB, and GVE in the genus *Vitivirus*, family *Betaflexiviridae*.

*P. vitis* was identified as a vector of only GLRaV−3 by Belli et al. [14]. The related species, the cottony maple scale, *Neopulvinaria innumerabilis* (Rathvon), was shown to transmit GLRaV−1, −3, and GVA [15,16]. Another species, the cottony maple leaf scale, *Pulvinaria acericola* (Walsh and Riley), is suspected to be a vector of GLD in the New York State’s Finger Lakes region [17]. To determine the vector ability of *P. vitis* in the east of France, we performed transmission experiments with populations from several vineyards, using two approaches: (1) infectivity assays, where natural populations were sampled in infected vineyards and transferred onto healthy recipient vines in the laboratory, and, (2) controlled transmission experiments, where newborn, thus nonviruliferous nymphs, were fed on infected leaves in the laboratory prior to their transfer onto healthy vines.

## 2. Materials and Methods

### 2.1. Origin of Viruses and Insects

For the infectivity experiments, *P. vitis* nymphs were collected between June and October in commercial vine plots located at Bennwihr, Kienheim, Nothalten and Turckheim, in Alsace, eastern France from 2004 to 2020. Grapevines bearing soft scales were analysed by ELISA (see Section 2.5 below) for GLRaV−1, −2, −3, and GVA before sampling insects. They were infected with GLRaV−1, −2 and −3, either alone or in various combinations, often with GVA (Table 1).

For controlled transmission tests, females with an ovisac were collected on pinot noir plots at Bennwihr (Table 1), Furdenheim (48°36′01.9″ N 7°32′39.4″ E), and Kienheim (48°41′43.1″ N 7°35′39.7″ E), and on a riesling vine plot at Nothalten (Table 1). Females were placed individually into 1.5 mL Eppendorf tubes, with a lid drilled with tiny holes for aeration.

### 2.2. Recipient Grapevines

Virus-free grapevines were issued either from rooted cuttings of the *Vitis vinifera* cvs. pinot noir clone P115, cabernet sauvignon or Syrah, issued from mother-stocks maintained under greenhouse, or from germinated seeds of pinot noir, pinot blanc, or Muscat Ottonel. These cuttings and seedlings were taken and germinated, respectively, in spring, and grown individually in pots under greenhouse, and were destined to be used as recipient plants at the 6–12 leaf stage. They were sprayed bi-monthly with an insecticide until one month before inoculation to avoid infestation by insects. In addition, a sample of these plants was tested before the transmission experiments by ELISA (see Section 2.5 below) to verify the absence of viruses (GLRaV−1, −3, −4, and GVA).

### 2.3. Infectivity Experiments

Nymph-bearing leaves were collected from leafroll-infected grapevines in vineyards, then fragments with nymphs from several leaves were cut out with scissors and clipped onto the leaves of healthy potted recipient vines. After a few days, the insects crawled off as the fragments dried. Two cuttings were inoculated with L1 nymphs, three with second instar nymphs (L2), and thirty with third instar nymphs (L3).

### 2.4. Controlled Transmission Experiments

Virus source grapevines were rooted cuttings from the following accessions of our reference collection of grapevine viruses [18]: P70 [19,20], Y199, Y217, Y252, Y258, Y318, and Cab119-66. Their virus content was checked by ELISA (see Section 2.5 below) and RT-PCR, as previously detailed in [21].

- First instar nymphs (L1)

Excised leaves of source grapevines were laid individually on their upper side into tight round polystyrene crystal boxes (15 or 17 cm diameter). A small wet piece of cotton was wrapped around the petiole of each leaf, then swathed tightly inside Parafilm^TM^ to retain water. After hatching inside Eppendorf tubes, crawlers were scattered onto these leaves for the acquisition access period (AAP). After the AAP, from 5 to 13 days, 2–3 leaf pieces from several leaves of source plants, with in total 50–100 L1 nymphs, were clipped onto leaves of potted recipient vines for a 5–9 days inoculation access period (IAP).

To make sure that the newly hatched crawlers were able to feed rapidly, we fed a sample of them on purified GVA virions, obtained following a previously described protocol [20], and added an equal volume of 60% sucrose solution with 1 µL methylene blue. A 90 µL drop of the solution was placed on a Parafilm^TM^ membrane stretched over the aperture of a black photo film box containing the crawlers. A second membrane was stretched over the drop, then the box was turned upside down to facilitate acquisition. The nymphs were allowed to feed on the membrane for 48 h. After the AAP, GVA detection by RT-PCR was performed on the purification solutions, on three batches of 10, 20, and 30 nymphs, and on a batch of 30 non-feeding nymphs collected on the box wall.

- Second instar nymphs (L2)

Females with ovisacs were placed directly on infected source leaves and crawlers were allowed to hatch and settle on the leaves. Between 14 days and 2 months of the AAP, leaves with L2 nymphs were cut and clipped onto the leaves of healthy recipient cuttings, as for the infectivity tests.

For accession Y318, 20 L2 and their honeydew were collected on a source vine at the end of the AAP to detect viruses by RT-PCR.

- Inoculation access period (IAP)

During the IAP, each recipient vine was isolated from the others under a 0.1 mm mesh micro-perforated plastic bag (‘bread bags’, Sealed Air SAS, Epernon, F). At the end of the IAP, the number of nymphs fixed on leaves or on the stem was counted. Then, recipient plants were sprayed with mevinphos (4 mL/l Phosdrin W10™) or chlorpyrifos-methyl (2 mL/l Reldan™) to kill the remaining insects. After two days, the sprayed plants were checked for any surviving insects, then transferred under a glasshouse compartment dedicated to recipient plants only. Cuttings with nymphs from uninfected grapevines were used as negative controls under the same conditions. All recipient plants were periodically sprayed with insecticide and fungicide and pruned to avoid overgrowth until the end of the study.

### 2.5. Virus Detection

Detection tests by ELISA were first performed on the leaves of the source vines, to verify the presence of viruses, and on a sample of recipient vines, prior to their inoculation in infectivity or transmission experiments, to verify their health status. Virus detection tests by ELISA on recipient vines were first undertaken 4–6 months after the IAP. After a dormancy period under unheated glasshouse conditions, plants were tested again 10–14 months after the IAP. Polyclonal antibodies produced in our laboratory were used for the detection of GLRaV−1, −2, −3, −4 strain 5, and GVA, in a biotin-streptavidin procedure [22]. Detection of GLRaV-4 strains 6 and 9 was performed using a commercial antiserum for GLRaV−4 strains 4–9 (Bioreba AG, Reinach, Switzerland) following the manufacturer’s instructions. Virus source grapevines and healthy cuttings or seedlings were used as positive and negative controls respectively. Samples that tested positive for viruses on at least two collection dates were considered positive. In addition, ELISA-positive plants were confirmed through RT-PCR. Viral RNA was extracted from plant material, then amplified with RNeasy™ Plant Mini Kit (Qiagen, Courtabœuf, France) using multiplexed reactions to search for GLRaV−1, −2, −3, and GVA following the protocol developed by [23].

## 3. Results

### 3.1. Infectivity of Natural Populations

*Pulvinaria vitis* nymphs sampled on infected vineyard plants were transferred onto healthy potted vines to evaluate their viral infectivity. Nymph numbers varied between 1 and 26 per recipient plant (*n* = 35, mean ± sd = 5.0 ± 5.2). At the end of the IAP, the number of nymphs fixed on recipient plants decreased considerably (0 to 6, mean ± sd = 1.4 ± 1.5). Of 35 recipient vines that received insects, over all of the sampling plots, only one transmission event was recorded, i.e., GLRaV−1 to one healthy cutting (P115) with L2 nymphs from the Kienheim Pinot gris plot (Table 1). This low rate of transmission could be due to the low number of nymphs settling on cuttings, to various viral molecular features in the source plots, and/or to a low vector competency of *P. vitis*. Moreover, possible genetic variations may exist between *P. vitis* populations and/or between virus strains infecting their host vines. Whatever the plot sampled, the source vine cultivar, or the recipient cultivar, GLRaV−3 and GVA were not transmitted to healthy vines. L3 nymphs showed poor settling behavior, which could explain why they transmitted no virus in our infectivity tests. Feeding records over 24 h by electropenetrography (EPG), using L3 nymphs placed on potted vines in autumn, showed that, out of 39 nymphs, 26 reached the xylem, and only one, the phloem (data not shown). L2 nymphs produced sometimes abundant honeydew during the AAP (Figure 1c), showing that they were feeding on phloem sap.

### 3.2. Controlled Transmission Experiments

Recipient vines that did not survive beyond 9 months for the second ELISA tests were discarded, which explains the limited number of plants for some transmission tests. Of 50 to 100 crawlers transferred after the AAP onto recipient vines, a mean of only 5.1 ± 6.1 were found settled on the latter at the end of the IAP. The majority of crawlers failed to settle and dehydrated to death.

**Figure 2:**
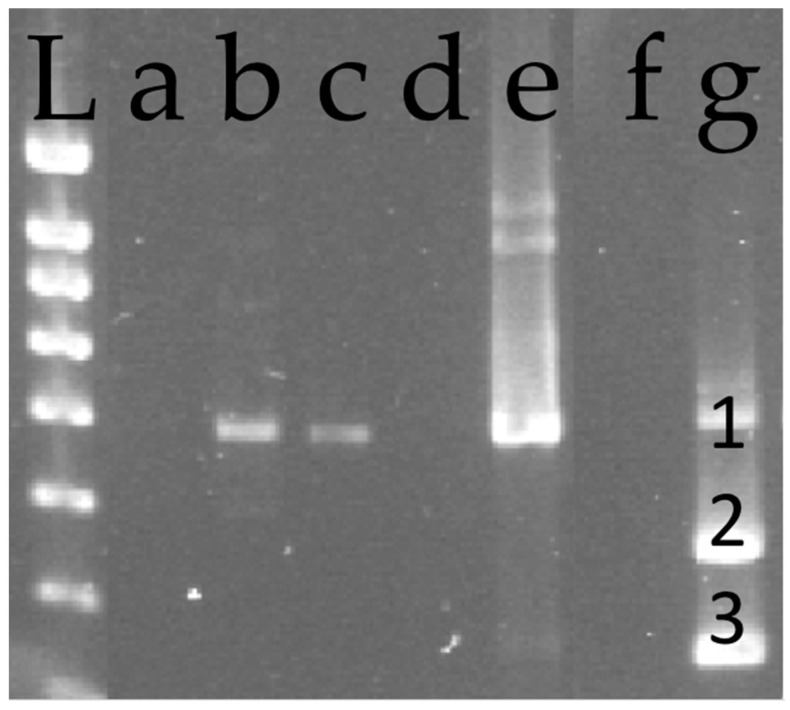
Agarose gel electrophoretic analysis of DNA fragments amplified from *Pulvinaria vitis* crawlers fed with purified GVA virions by reverse transcription-polymerase chain reaction (RT-PCR). L: 100-bp DNA ladder; lanes a to c: batches of *P.* crawlers fed on purified GVA virions collected on Parafilm^TM^ membrane; lane a: batch of 10 crawlers; lane b: batch of 20 crawlers; lane c: batch of 30 crawlers; lane d: batch of 30 crawlers non feeding on purified GVA virions and collected from the box wall; lane e: purified GVA virions; lane f: healthy control = water with primers; lane g: leaf extract of the accession Y258 used as positive control for GLRaV−1, GLRaV−3 and GVA; 1 = GVA band (524 bp), 2 = GLRaV−1 band (382 bp), 3 = GLRaV−3 band (282 bp).

A membrane feeding assay with purified GVA virions added and methylene blue showed the ability of crawlers to feed across the membrane. After 48 h AAP on the membrane, the gut of crawlers that fed was stained blue (Figure 1b). In addition, GVA was detected by RT-PCR in the feeding medium (Figure 2, lane e) and, after 48 h AAP, in batches of 20 and 30 crawlers fed on purified GVA virions added with sucrose (lanes b and c), but neither in that of 10 crawlers that settled on the membrane (lane a), or in that of crawlers collected from the box wall (lane d).

While the number of L2 nymphs deposited per recipient vine averaged 16.3 ± 11.4, only 4.4 ± 3.6 of them (27.0%) were found settled on leaves at the end of the IAP. Of four different populations, nymphs from two vineyards (Table 2) were able to transmit GLRaV−1 and GVA, but not GLRaV−3, to recipient vines. Moreover, GLRaV−1, −3, and GVA were detected by RT-PCR in a 20 L2 nymph batch and in their honeydew collected on the source vine Y318. Positive detection of viruses showed that nymphs fed on source vine thus ingested and excreted virions, but no transmission event occurred from this accession (Table 2).

In our assays, *P. vitis* was unable to transmit the different GLRaV−4 strains 4, 5, 6, and 9 tested. For this reason, we did not test for GVA which was associated with these strains in the source plants.

## 4. Discussion

We report here, for the first time, that the cottony grape scale, *Pulvinaria vitis*, is a vector of two widespread grapevine-infecting viruses, GLRaV−1 and GVA, while this species was previously only known as a vector of GLRaV−3 in Italy [14]. This ability was demonstrated by means of both (1) infectivity tests, i.e., using nymphs sampled from virus-infected vineyards and transferred onto healthy vines for inoculation, and, (2) controlled transmission tests, i.e., using laboratory-borne nymphs submitted to a controlled acquisition–inoculation process. Infectivity experiments yielded only one transmission event with GLRaV−1; however, the viral populations may have been different among the various source plots, in vector availability, in molecular features, and/or other factors. In contrast, our controlled transmission experiments, provide evidence that *P. vitis* can transmit GLRaV−1 and GVA from vine to vine. Since it is also a GLRaV−3 vector [14], *P. vitis* can thus transmit the same three grapevine-infecting viruses as the closely related soft scale species *N. innumerabilis* [15,16]. Moreover, our results show that *P. vitis* L1 and L2 nymphs, but probably not the less active L3 nymphs, could play a role in transmission and dispersal of grapevine ampelo- and vitiviruses in vineyards, beside other better-known and more efficient scale insect species, mainly mealybugs [13]. Moreover, *P. vitis* was able to transmit GVA, along with GLRaV−1, from pinot noir accession P70, as did another soft scale insect, *Parthenolecanium corni* (Bouché), the European fruit lecanium [24]. As for the latter species, *P. vitis* females produce a great number of crawlers which may be carried away by the wind [5,25] and are thus able to land on new grapevine plots. Since *P. vitis* nymphs are often scarce and scattered throughout the foliage, the potential risk of contamination by this species is probably underestimated. Further work is anticipated to better assess the role of *P. vitis* in the natural spread of GLD alongside other species.

## Figures and Tables

**Table 1:** Location of the plots sampled for *Pulvinaria vitis* nymphs and number of recipient vines inoculated in infectivity tests according to virus content of source vines.

	Locality	Bennwihr	Kienheim	Kienheim	Nothalten	Turckheim
	Latitude	48°08′18″ N	48°41′33″ N	48°41′37″ N	48°21′32″ N	48°05′42″ N
	Longitude	7°19′07″ E	7°35′32″ E	7°35′33″ E	7°24′40″ E	7°16′34″ E
	Variety	Pinot noir	Gewurztraminer	Pinot gris	Riesling	Sylvaner
source vine viruses	GLRaV−1		1	1	3	
	GLRaV−3	3				
	GLRaV−1; GVA				9	1
	GLRaV−1,−3	1			2	
	GLRaV−3; GVA				1	
	GLRaV−1,−3; GVA	2			10	
	GLRaV−1,−2,−3; GVA				1	
	number of vines tested	6	1	1	26	1

**Table 2:** Outcomes of transmission experiments performed with L1 and L2 nymphs of *Pulvinaria vitis*, according to nymph origin, cultivar, and virus association in source vine. Transmission rate = number of positive plants/number of inoculated plants, nt = not tested. Transmission events are in bold.

		Cultivar and Accession			Transmission Rates							
Soft Scale	Mean No.		Vine	Viruses in	GLRaV−1	GLRaV−3	GLRaV−4 Strains				GVA	Test
Origin	/Stage	for AAP	Origin	Source Vines			4	5	6	9		Vines
Kienheim	100 L1	Liali Bidona Y258	Armenia	GLRaV−1, −3; GVA	**3/4**	0/4	-	-	-	-	**1/4**	P115
Nothalten	18 L2	Yshouhali Y318	Iran	GLRaV−1, −3; GVA	0/10	0/10	-	-	-	-	0/10	P115
Bennwihr	50 L1	Pinot noir P70	France	GLRaV−1; GVA	**2/4**	-	-	-	-	-	0/4	P115
Furdenheim	12 L2	Pinot noir P70			0/10	-	-	-	-	-	0/10	P115
Kienheim	23 L2	Pinot noir P70			**7/13**	-	-	-	-	-	**5/13**	P115
Nothalten	100 L1	Koudsi Y252	Israel	GLRaV−4; GVA	-	-	0/10	-	-	-	nt	MO
Nothalten	100 L1	Black Monukka Y199	USA	GLRaV−5, −6; GVA	-	-	-	0/11	0/11	-	nt	MO
Nothalten	100 L1	White Emperor Y217	USA	GLRaV−5; GVA	-	-	-	0/10	-	-	nt	MO
Nothalten	100 L1	Cabernet Sauvignon Cab 119-66	France	GLRaV−9; GVA	-	-	-	-	-	0/11	nt	MO

## Data Availability

Data supporting reported the results are available on request.
